# Ecological analyses of the associations between injury risk and socioeconomic status, geography and Aboriginal ethnicity in British Columbia, Canada

**DOI:** 10.1186/s40064-016-2262-x

**Published:** 2016-05-10

**Authors:** M. A. George, M. Brussoni, A. Jin, C. E. Lalonde, R. McCormick

**Affiliations:** Department of Pediatrics, Faculty of Medicine, University of British Columbia, Vancouver, BC Canada; Child and Family Research Institute, Room F508, 4480 Oak Street, Vancouver, BC V6H-3V4 Canada; School of Population and Public Health, University of British Columbia, Vancouver, BC Canada; Epidemiology Consultant, Surrey, BC Canada; Department of Psychology, Faculty of Social Sciences, University of Victoria, Victoria, BC Canada; Faculty of Human, Social and Educational Development, Thompson Rivers University, Kamloops, BC Canada

**Keywords:** Aboriginal, Indigenous populations (MeSH), Indians, North American (MeSH), First Nations, Medical record linkage (MeSH), Hospitalization (MeSH), Injury and wounds (MeSH), Epidemiology (MeSH)

## Abstract

**Background:**

The current study examines what factors contribute to higher injury risk among Aboriginal peoples, compared to the total British Columbia (BC) population. We explore socioeconomic, geographic, and cultural factors, and combinations of these factors, that contribute to increased injury risk for Aboriginal peoples. This follows from our previously reported findings of improvements in injury risk over time for both the total and Aboriginal populations.

**Data and methods:**

We use provincial population-based linked health care databases of hospital discharge records. We identify three population groups: total BC population, and Aboriginal populations living off-reserve, or on-reserve. For each group we calculate age and gender-standardized relative risks (SRR) of injury-related hospitalization, relative to the total population of BC, for two 5-year time periods (1999–2003, and 2004–2008). We use custom data from the 2001 and 2006 long-form Censuses that described income, education, employment, housing conditions, proportion of urban dwellers, proportion of rural dwellers, and prevalence of Aboriginal ethnicity. We use multivariable linear regression to examine the associations between the census characteristics and SRR of injury.

**Results:**

The best-fitting model was an excellent fit (R^2^ = 0.905, p < 0.001) among the three population groups within Health Service Delivery Areas of BC. We find indicators in all three categories (socioeconomic, geographic, and cultural) are associated with disparity in injury risk. While the socioeconomic indicators (income, education, housing, employment) were shown to be highly correlated, only living in housing that needs major repair and occupational hazardousness, along with rural residence and Aboriginal ethnicity, remained in the final model. Our data show that cultural density is not associated with injury risk for Aboriginal peoples, and that living off-reserve is associated with reduced injury by improving socioeconomic and geographic conditions (compared to living on-reserve). Finally, our analyses show that Aboriginal status itself is associated with injury risk.

**Conclusions:**

Our findings confirm previous research indicating that geographical differences differentiate injury risk, including for Aboriginal populations, and that socioeconomic determinants are associated with health risks. Our analyses showing that Aboriginal status itself contributes to injury risk is new, but we can only speculate about pathway, and whether the causes are direct or indirect.

## Background

Indigenous peoples are among the poorest, least educated and least healthy of Canadians (Kolahdooz et al. 2015; CCPA 2010; Adelson 2005; RCAP 1996). Improving the health of Indigenous Canadians is a critical but complex challenge (Stephens et al. 2005).

Internationally, evidence suggests an association between health disparities and social determinants of health (WHO 2011; Marmot and Wilkinson 2005). Income inequality has been well-documented as a determinant of poor health (Coburn 2004; Lynch et al. 2004a, b; Ross et al. 2000; Kosteniuk and Dickinson 2003; Wilkinson 1996; Kawachi et al. 1997). Increasing income will reduce barriers to accessing resources, including adequate food and health care for the most disadvantaged, yet may not be sufficient in reducing health inequalities (House and Williams 2003).

Income disparities are a product of national and regional background and historical factors (Lynch 2003). Every nation has its own social, political and economic history that contributes to its inequalities in both income and health, with organizational and political structure, and social positioning within that structure, being potential causal pathways to health status (Ross et al. 2006).

Health outcomes are tied to social positioning, so that those in the lowest categories of income, when comparing states or countries, are less healthy than those in the next lowest category, and so on up to the highest income categories (Ross et al. 2006). In the United States (US), it has been shown that this is case for both within and between White and African American populations. Within each income category, Whites have better health than African American people, yet the socioeconomic status (SES) difference within each racial group is larger than the differences among groups (House and Williams 2003). Importantly, when SES is controlled in statistical models, an independent effect of race has been observed, so that at every income level, African-American populations have lower levels than Whites for life expectancy, and infant mortality (House and Williams 2003). Kawachi et al. (2005) argue that historical, political and ideological barriers in the US have hindered analyses of race and class as co-determinants of health, and that race is not merely a proxy for socioeconomic determinants.

Previously, we reported disparities in injury risk between Aboriginal and total British Columbia (BC) populations for all injuries, and that time trends indicated a narrowing gap for all injuries combined (George et al. 2015a; Jin et al. 2015a), including among children and youth under age 25 (George et al. 2015b). By category of injury, the gap risk has narrowed for unintentional falls (Jin et al. 2015b) but not for iatrogenic injuries (medical/surgical mishaps) (Jin et al. 2016). Within the Aboriginal populations in BC, results indicated that living in remote areas and not having completed high school were associated with increased risk of injury (Brussoni et al. 2014) and that risk varied according to economic conditions for workplace injuries (Jin et al. 2014).

In the current study, we investigate socioeconomic, geographic, and cultural factors, as well as combinations of these factors that contribute to increased risk of injury for Aboriginal peoples, compared to the general population.

## Methods

The University of British Columbia Behavioural Research Ethics Board reviewed and approved our methods (BREB file H06-80585). The Data Stewards representing the BC Ministry of Health and the BC Vital Statistics Agency approved the data access requests. We used existing databases, permanently linked by British Columbia Personal Health Number, maintained by Population Data BC (Canadian Institute for Health Information 2012; British Columbia Ministry of Health. MSP 2012; British Columbia Vital Statistics Agency. Births 2011a; Deaths 2011b). Population Data BC rendered the client records anonymous before our analysis.

### Population counts

For purposes of calculating denominator populations for injury hospitalization rates, we used the registration and premium billing files (British Columbia Ministry of Health. MSP 2012) of the Medical Services Plan of BC (MSP, the province’s universal health care insurance program) as a population registry, counting the total resident population of BC at the mid-points of fiscal years 1998–1999 through 2008–2009. Within this population, we defined as “Aboriginal” any person with: (a) membership in MSP Premium Group 21 (indicating insurance premiums paid by First Nations and Inuit Health Program, Health Canada, for reason of Indian status, as defined by the Indian Act of Canada), or (b) one or both parents with Indian status or resident on an Indian Reserve, as indicated on the linked Vital Statistics birth record (British Columbia Vital Statistics Agency. Births 2011a), or (c) Indian status or resident of an Indian Reserve, as indicated on the linked Vital Statistics death record (British Columbia Vital Statistics Agency. Deaths 2011b). We previously described this method, and discussed the quality of the population registry, and validity and limitations of the Aboriginal identification (George et al. 2015a; Jin et al. 2015a).

Within the population registry, we classified as “on-reserve” those Aboriginal people residing in a postal code area associated with an Indian reserve or settlement recognized by Statistics Canada and the federal Department of Aboriginal Affairs and Northern Development. We classified all other Aboriginal people as “off-reserve”.

There are sixteen Health Service Delivery Areas (HSDAs) in BC. We tabulated population counts by calendar year (three quarters of the starting fiscal year plus one quarter of the ending fiscal year), gender, 5-year age group, Aboriginal status, community, reserve residence, and HSDA.

### Hospitalization counts

 We tabulated counts of hospital separations (Canadian Institute for Health Information 2012) among residents of BC, occurring from January 1, 1999 through December 31, 2008. We considered a hospitalization as “due to injury” if the level of care was “acute” or “rehabilitation”, and the Most Responsible Diagnosis on the discharge record was an International Classification of Diseases Revision 9 (“ICD-9”) numeric code in the range 800 through 999, or an International Classification of Diseases Revision 10 (“ICD-10”) code in the range S00 through T98.

Linking individual hospitalization records to the population registry, we tabulated counts of hospitalizations due to injury by calendar year, gender, 5-year age group, Aboriginal status, reserve residence, and HSDA.

### Risk of hospitalization

As in previously reported analyses (Brussoni et al. 2014, 2016; Jin et al. 2014, 2015a, b; George et al. 2015a, b), we calculated standardized relative risk (SRR) of hospitalization relative to the risk of hospitalization in the reference population (the total population of BC) using the method of indirect standardization (Kahn and Sempos 1989), standardizing by gender and 5-year age group. The SRR is analogous to the Standardized Mortality Ratio (if death is the event counted), and could also be called the Standardized Incidence Ratio.

### Predictors of risk

We studied risk markers for hospitalization due to injury, using an ecological approach, where the unit of observation was the HSDA (n = 16) subdivided into three population groups (total population, Aboriginal off-reserve, and Aboriginal on-reserve) and two time periods (1999–2003, and 2004–2008). Two of the 16 HSDAs contained no Indian reserves, so the total number of observation units was 92 [14 HSDAs × 3 populations plus 2 time periods × 2(×2)]. The total population includes the two Aboriginal subpopulations, but we corrected the effect by including the proportion of the population who are Aboriginal as an independent variable in the multivariable analysis (see below). As hypothesized risk markers, we selected socio-economic, housing, and geographic indicators previously developed by Statistics Canada and Aboriginal Affairs and Northern Development Canada. We previously published definitions and reasons for selection of the markers (Brussoni et al. 2014, 2016; Jin et al. 2014, 2015a, b; George et al. 2015a, b).

From the 2001 and the 2006 long form Censuses of Canada, we measured the following hypothesized socio-economic markers of injury risk, for each HSDA sub-population: (1) Total (annual) Income per capita, (2) Community Well-Being Income Score (i.e., total annual income per capita, logarithmically scaled) (Penney et al. 2012), (3) proportion of population, age 25+ years with at least a high school completion certificate, (4) proportion of population, age 25+ years with university degree, bachelors or higher, (5) average population per room (an index of the degree of crowding in the population’s housing), (6) proportion of the population living in a dwelling in need of major repair, (7) proportion of population, age 25+ years, in the labour force, (8) proportion of population, age 25+ years, employed (for pay), (9) proportion of population who identified themselves as “an Aboriginal person, that is, North American Indian, Métis or Inuit (Eskimo)”, (10) proportion of population who gave only one response to the ethnic origin question, and it was a group that could be classified as North American Indian, (11) proportion of the HSDA’s population classified as “urban” (residing in a population centre with 100,000 or more persons), and (12) proportion of the HSDA’s population classified as “rural” (residing in a population centre with fewer than 1000 persons, or in an area with population density <400 persons per km^2^).

We obtained the housing indicators (5 and 6 above) by requesting a custom tabulation that linked dwellings to individuals in a relational database, with the universe of individuals as the index file, and the universe of dwellings as the keyed look-up table. Each dwelling is represented in the population statistics as many times as it has occupants. The housing indicators are weighted by population, not by number of dwellings, thus these indicators represent the living conditions of the population.

We calculated the following work-related statistics of injury risk, relative to the population of BC: (13) relative risk of work injury compensation claim, expected from occupational categories, and (14) relative risk of work injury compensation claim, expected from industry categories. We defined these two markers as in a previous report focused on worker compensation injuries. These two markers summarize the hazardousness of the distribution of the labour force among occupational and industrial categories. We also created four interaction variables, calculated as each of the employment-related risk markers (13 and 14 above) multiplied by the proportion of the population who were employed, and by the proportion who were in the labour force.

### Ecological analysis

For each HSDA sub-population, we calculated the age and gender standardized SRR of hospitalization due to injury during the period 1999 through 2003 (a 5-year period centred about the Census year 2001) and during the period 2004 through 2008 (centred about the Census year 2006), relative to the total population of BC during the same time period. The distribution of the SRRs was approximately normal (Kolmogorov–Smirnov statistic 0.058, df = 92, p = 0.200, Shapiro–Wilk statistic 0.972, df = 92, p = 0.044); therefore we started with SRR untransformed as the dependent (Y) variable for regression analysis.

We tested hypotheses of association by performing least-squares linear regressions. We tested census year, hypothesized socio-economic, work-related and geographic markers, in turn as the single independent variable. Variables that had statistically significant association (p < 0.05) with SRR of worker compensation injury in univariate analysis were included in subsequent multivariable regression analysis. Beginning with the variable most strongly correlated with SRR (largest coefficient of determination R^2^ in the univariate analysis), but excluding the Aboriginal identity and North American Indian ethnic origin variables), we used stepwise forwards addition of variables to arrive at the best-fitting multivariable model. At each step, the variable with the largest *p* value >0.05 was eliminated. Addition and elimination stopped when all independent variables had regression coefficients significantly different from zero (p < 0.05) and the list of candidate variables was exhausted. At that point we introduced the ethnicity variables, and determined the best-fitting model that also included ethnicity. In the final model, we tested the normality of the distribution of the standardized residuals by the Kolmogorov–Smirnov and Shapiro–Wilk statistics, and we verified homoscedasticity by scatter-plotting the standardized residuals against the regression-predicted values of SRR.

The regression coefficient (“B”) of each independent variable represents the change in the dependent variable SRR that is associated with a unit change in the independent variable. The absolute change in SRR associated with a change of one standard deviation (SD) in the independent variable is calculated as *B* × *SD*. Repeating the calculation with the lower and upper 95 % confidence limits of B gives the confidence limits of the change in SRR.

Given that the regression was weighted by population, and the Aboriginal off-reserve and Aboriginal on-reserve population units were much smaller than the total populations of HSDAs, it was necessary to verify that the step-wise regression procedure had indeed produced a model representative of the experience of the Aboriginal populations. This was done by using the final regression model as a risk prediction calculator, then comparing the predicted disparities of injury SRR among the three population groups (total population, Aboriginal off-reserve, and Aboriginal on-reserve) to the observed disparities among the three groups (with all HSDAs combined).

## Results

Table 1 provides descriptive data for each variable, by two time periods, for the three populations: total BC, Aboriginal living off-reserve, and Aboriginal living on-reserve. In both time periods (1999–2003 and 2004–2008) the total population’s SRR of injury is one, because SRR is relative to the total population of BC *during the same period*. This removes the effect of the secular trend of declining injury risk in BC (George et al. 2015a; Jin et al. 2015a).

**Table 1:** Descriptive profile of three population groups in British Columbia

Variable description	Period	Population		
		Total population	Off-reserve Aboriginal	On-reserve Aboriginal
Age and gender-standardized relative risk of hospital separation due to all injury	1999–2003	1	2.43	2.91
	2004–2008	1	2.38	2.77
Person-years of observation	1999–2003	20,663,214	363,704	301,529
	2004–2008	21,916,203	431,968	308,371
Mean annual person count	1999–2003	4,132,643	72,741	60,306
	2004–2008	4,383,241	86,394	61,674
Census total population	2001	3,868,875	123,640	46,385
	2006	4,074,380	145,020	51,060
Total income per capita	2001	$22,890	$13,357	$9994
	2006	$27,370	$16,619	$10,797
CWB income score	2001	81.4	63.4	53.7
	2006	87.3	70.7	56.3
Proportion of population, age 25+ years with at least a high school certificate	2001	0.720	0.590	0.496
	2006	0.834	0.716	0.530
Proportion of population, age 25+ years with university degree, bachelors or higher	2001	0.161	0.049	0.020
	2006	0.217	0.079	0.035
Average number of persons per room	2001	0.478	0.547	0.683
	2006	0.471	0.522	0.677
Proportion of population residing in dwelling requiring major repairs	2001	0.083	0.159	0.343
	2006	0.074	0.149	0.390
Proportion of population, age 25+ years, labour force participation	2001	0.658	0.677	0.641
	2006	0.658	0.701	0.616
Proportion of population, age 25+ years, employed	2001	0.611	0.549	0.470
	2006	0.624	0.626	0.476
Risk of work injury claim, relative to BC pop 2006, expected from occupation, labour force aged 15+ years	2001	0.992	1.161	1.127
	2006	1.000	1.191	1.143
Risk of work injury claim, relative to BC pop 2006, expected from industry, labour force aged 15+ years	2001	1.008	1.094	1.077
	2006	1.000	1.107	1.086
Proportion of population, Aboriginal identity	2001	0.044	1.000	1.000
	2006	0.048	1.000	1.000
Proportion of population, North American Indian single response	2001	0.031	0.600	0.950
	2006	0.032	0.554	0.965
Proportion of HSDA population residing in large urban population centre	2001	0.608	0.375	0.216
	2006	0.616	0.371	0.216
Proportion of HSDA population residing in rural area	2001	0.145	0.231	0.292
	2006	0.142	0.232	0.290

By 2006, 4.8 % of the 4.1 million total BC population identified as being Aboriginal, with 3.6 % living off-reserve and 1.2 % living on-reserve. Per capita income and education achievement were both higher for the total BC population than for the Aboriginal population, and within the latter, for those living off-reserve compared to living on-reserve. Housing conditions were less favourable for Aboriginal peoples compared to the total BC population when measured by the proportion of people living in one house and for houses needing major repair.

A higher proportion of the total BC population lived in large urban centres, compared to Aboriginal peoples. Higher proportions of the Aboriginal populations living off-reserve lived in large urban centres than those living on-reserve.

Aboriginal peoples living off-reserve had the highest participation rate in the labour force, followed by the total BC population, and the Aboriginal population living on-reserve. Compared to the total BC population, employment-related injury risk was higher for Aboriginal peoples, measured by proportions of the labour force working in occupations or working in industries with increased risk of compensation claims.

Table 2 shows results from linear regression models testing one independent (X) variable as a predictor of injury SRR (the Y variable). As expected, most of the hypothesized predictor variables were statistically significantly associated with injury risk, and were retained for subsequent step-wise multivariable analysis.

**Table 2:** Ecologic analysis of risk of hospitalization due to injury among Health Service Delivery Area population groups in British Columbia, 1999–2008*

X variable	Min	Max	Mean^‡^	SD^‡^	N	R^2^	B^§^	SE^¶^	p^\\^	SRR change per SD**	L95CL^††^	U95CL^‡‡^
*Regression* ^†^ *statistics from models with one independent (X) variable*												
Census	2001	2006	2003.5	2.5	92	0.000	−0.001	0.016	0.946	−0.003	−0.084	0.079
Income per capital	7.7	36.0	17.1	6.4	92	0.296	−0.050	0.008	0.000	−0.318	−0.420	−0.215
Income score	45.1	96.5	69.5	12.4	92	0.403	−0.039	0.005	0.000	−0.488	−0.613	−0.364
High school	0.315	0.907	0.650	0.132	92	0.345	−2.787	0.405	0.000	−0.369	−0.476	−0.263
University degree	0.000	0.364	0.084	0.076	92	0.400	−2.816	0.363	0.000	−0.213	−0.268	−0.158
Population per room	0.403	0.812	0.549	0.097	92	0.000	0.029	0.792	0.971	0.003	−0.150	0.156
House needs major repairs	0.050	0.478	0.186	0.116	92	0.591	7.215	0.633	0.000	0.838	0.691	0.984
Labour force	0.515	0.771	0.664	0.053	92	0.004	−0.604	1.062	0.571	−0.032	−0.145	0.080
Employed	0.380	0.734	0.572	0.083	92	0.218	−4.226	0.844	0.000	−0.352	−0.491	−0.212
Occupation risk	0.805	1.446	1.111	0.146	92	0.376	1.636	0.222	0.000	0.240	0.175	0.304
Industry risk	0.687	1.258	1.064	0.108	92	0.305	1.977	0.314	0.000	0.214	0.146	0.281
Occupation risk employed	0.350	0.934	0.635	0.126	92	0.130	1.526	0.416	0.000	0.192	0.088	0.295
Industry risk employed	0.299	0.826	0.609	0.113	92	0.049	1.186	0.552	0.034	0.134	0.010	0.257
Occupation risk labour force	0.510	1.055	0.739	0.124	92	0.306	2.064	0.328	0.000	0.255	0.175	0.336
Industry risk labour force	0.448	0.900	0.708	0.102	92	0.220	2.236	0.444	0.000	0.228	0.138	0.318
Urban	0.000	1.000	0.386	0.416	92	0.449	−0.658	0.077	0.000	−0.274	−0.337	−0.210
Rural	0.000	0.446	0.228	0.153	92	0.482	1.889	0.207	0.000	0.289	0.226	0.351
Aboriginal	0.007	1.010	0.676	0.447	92	0.690	1.853	0.131	0.000	0.829	0.712	0.945
North American Indian	0.004	0.992	0.501	0.377	92	0.683	2.336	0.168	0.000	0.881	0.755	1.006

* Three population groups (total, Aboriginal on-reserve and Aboriginal off-reserve) divided by 16 HSDAs and 2 time periods (1998–2003 and 2004–2008)

^†^The dependent (Y) variable is the standardized relative risk (SRR) of hospitalization due to injury, and regression is weighted by person-years

^‡^Unweighted mean and standard deviation (SD) of the independent (X) variable

^§^B = regression coefficient

^¶^SE = standard error of the regression coefficient

^\\^p = probability that B = 0

** One SD change in the independent variable is associated with this absolute change in the SRR of injury. E.g., one SD change in Income Per Capita ($6400) is associated with reduction in SRR of 0.318

^††^Lower limit of the 95 % confidence interval for the Relative Risk Ratio per SD

^‡‡^Upper limit of the 95 % confidence interval for the Relative Risk Ratio per SD

Table 3 shows the best-fitting regression model with multiple independent (X) variables. The best-fitting model was an excellent fit, explaining 90.5 % of the variance in injury risk among population groups within HSDAs (R^2^ = 0.905, p < 0.001). The standardized residuals appeared to be normally distributed (Kolmogorov–Smirnov statistic 0.075, df = 92, p = 0.200; Shapiro–Wilk statistic 0.990, df = 92, p = 0.699).

**Table 3:** Ecologic analysis of risk of hospitalization due to injury among Health Service Delivery Area population groups in British Columbia, 1999–2008*

X variable	Min	Max	Mean^‡^	SD^‡^	N	B^§^	L95CL	U95CL	SE^\\^	p**	SRR change per SD^††^	L95CL^‡‡^	U95CL^‡‡^
*Regression* ^†^ *statistics from best-fitting model with multiple independent (X) variables*													
(Constant)					92	0.321	0.029	0.614	0.147	0.032	0.000	0.000	0.000
House needs major repairs	0.050	0.478	0.186	0.116	92	1.769	0.661	2.877	0.557	0.002	0.205	0.077	0.334
Rural	0.000	0.446	0.228	0.153	92	0.954	0.652	1.256	0.152	0.000	0.146	0.100	0.192
Occupation risk	0.805	1.446	1.111	0.146	92	0.357	0.067	0.647	0.146	0.016	0.052	0.010	0.095
Aboriginal	0.007	1.010	0.676	0.447	92	1.169	0.911	1.426	0.129	0.000	0.523	0.408	0.638

Multivariable model statistics: R squared = 0.905, F = 208.254, p = 0.000

* Three population groups (total, Aboriginal on-reserve and Aboriginal off-reserve) divided by 16 HSDAs and 2 time periods (1998–2003 and 2004–2008)

^†^The dependent (Y) variable is standardized relative risk (SRR) of hospitalization due to injury, and regression is weighted by person-years

^‡^Unweighted mean and standard deviation (SD) of the independent (X) variable

^§^B = regression coefficient

^¶^95 % confidence limit for the Relative Risk Ratio per SD

^\\^SE = standard error of the regression coefficient

** p = probability that B = 0

^††^Relative Risk Ratio per SD = exp(BxSD). One SD change in the independent variable is associated with this absolute change in the SRR of injury

^‡‡^95 % confidence limit for the Relative Risk Ratio per SD

The best-fitting model retained the following variables as independent, statistically significant predictors of injury risk: (1) population proportion residing in a house needing major repairs: one standard deviation increase (0.116) was associated with absolute increase of 0.205 in the SRR of injury (p = 0.002); (2) population proportion residing in a rural area: one standard deviation increase (0.153) was associated with absolute increase of 0.146 in the SRR of injury (p < 0.001); (3) hazardousness of the labour force distribution among occupations: one standard deviation increase (0.146) was associated with absolute increase of 0.052 in the SRR of injury (p = 0.016); (4) population proportion self-identifying as Aboriginal: one standard deviation increase (0.447) was associated with absolute increase of 0.523 in the SRR of injury (p < 0.001).

Table 4 shows the absolute disparities between the three population groups predicted by the best-fitting multivariable regression model, combining both time periods (1999–2003 and 2004–2008). The model predicts that the Aboriginal off-reserve population will have SRR of injury 1.40 higher than the total population, and that the Aboriginal on-reserve population will have SRR of injury 1.82 higher than the total population. These predictions are very close to the observed disparities shown in Table 1: the Aboriginal off-reserve population had SRR 1.43 (i.e., 2.43 − 1) higher during the period 1999–2003, and 1.38 (i.e., 2.38 − 1) higher during the period 2004–2008 than did the total population (SRR = 1, by definition), and the Aboriginal on-reserve population had SRR 1.91 (i.e., 2.91 − 1) higher during the period 1999–2003, and 1.77 (i.e., 2.77 − 1) higher during the period 2004–2008 than did the total population.

**Table 4:** Disparities of injury risk predicted by the best-fitting multivariable regression model

Variable	Total population	Off-reserve Aboriginal					On-reserve Aboriginal				
	Mean*	Mean*	Difference^†^	SRR change^‡^	L95CL^‡^	U95CL^‡^	Mean*	Difference^†^	SRR change^‡^	L95CL^‡^	U95CL^‡^
Need major repairs	0.078	0.153	0.075	0.133	0.05	0.22	0.367	0.289	0.511	0.19	0.83
Rural	0.143	0.232	0.089	0.084	0.06	0.11	0.291	0.148	0.141	0.10	0.19
Occupation risk	0.996	1.178	0.181	0.065	0.01	0.12	1.135	0.139	0.050	0.01	0.09
Aboriginal	0.046	1.000	0.954	1.115	0.87	1.36	1.000	0.954	1.115	0.87	1.36
Total disparity (sum)				1.396					1.817		

* Population-weighted mean of the x-variable, 2001 and 2006 Census, for the specified population group

^†^Difference between mean of the specified population group and mean of the total population

^‡^Predicted absolute change in standardized relative risk of injury (calculated as B x difference), where B is the regression coefficient in the best-fitting multivariable model

The best-fitting regression model is additive (i.e., linear); therefore, Table 4 also allows us to proportionately allocate the increase in SRR, according to the estimated contribution of each independent variable to the total predicted disparity. Among the Aboriginal off-reserve population, 9.5 % of the disparity with the total population (i.e., 0.133/1.396) is attributable to housing condition, 6.0 % (i.e., 0.084/1.396) is attributable to rural residence, 4.6 % (i.e., 0.065/1.396) is attributable to occupational risk, and 79.8 % (i.e., 1.115/1.396) is attributable to Aboriginal ethnicity. Among the Aboriginal off-reserve population, 28.2 % of the disparity with the total population (i.e., 0.511/1.817) is attributable to housing condition, 7.8 % is attributable to rural residence, 2.7 % is attributable to occupational risk, and 61.4 % is attributable to Aboriginal ethnicity.

Since the off-reserve and on-reserve populations both consist entirely of people who self-identify as Aboriginal, the disparity between the two populations is fully attributable to the combined effects of housing condition, rural residence and occupational risk. The largest portion [90 %, i.e., (0.511–0.133)/(1.817–1.396)] is attributable to the much worse housing conditions on-reserve.

## Discussion, limitations and conclusions

Our ecological analyses examined the contributions of socioeconomic and cultural backgrounds and geography to injury risk. We have previously reported that geography is predictive of injury risk for Aboriginal populations of BC, with those living in major metropolitan areas having lower risk than those living in non-metropolitan areas—either on- reserve or off-reserve (Brussoni et al. 2014). Our current analyses found associations with disparity of injury risk and all three categories; that is, socioeconomic, cultural, and geographic. While the socioeconomic indicators (income, education, housing, employment) have been generally known, and specifically shown in our data, to be highly correlated, only one indicator in this category—housing needs major repair—remained in the final, best-fitting model. Housing condition may be a good summary indicator of socioeconomic conditions. At the simplest level, living in housing that needs major repair may pose physical hazards, and housing condition has been shown to be a mediating factor between poverty and falls, a major category of injury (Hu et al. 2015; Shenassa et al. 2004). On the other hand, housing conditions may be indicative of a lack of resources. At a more complex level, communities with high numbers of houses that need major repair may have issues signifying lack of response to needs from local, provincial or federal levels of government for fiscal or other reasons. Or, it may be that inequality, including as measured by housing conditions in a community, rather than poverty is associated with health disparity (Marmot and Wilkinson 2005; Shenassa et al. 2004).

We have previously reported that the risk of workplace injury among the Aboriginal population increases in accordance with employment rates (Jin et al. 2014). In our current analyses, we found occupational risk to be associated with higher risk of injury. Occupational risk may have a direct effect, it may be a proxy for socioeconomic disadvantage, or it may be an indicator of more generalized risk-taking behaviour.

Our data show that: (1) cultural density does not influence risk to Aboriginal peoples, (2) living off-reserve reduces risk by improving socioeconomic and geographic conditions, and (3) ethnicity itself is associated with most of the disadvantage suffered by Aboriginal peoples.

Aboriginal status has been cited as a determinant of health; however, in the literature, researchers frequently describe this as an *indirect* determinant (Wilkinson and Pickett 2009; Gracey and King 2009; King et al. 2009). In their recent systematic review of the literature on social determinants of health, Kolahdooz et al. (2015) found that the health of Aboriginal peoples was disproportionally affected by social determinants of health, although they found limited data on the associations between income, personal and social circumstances and health outcomes. They also found that the literature was sparse regarding the factors contributing to housing status and its impacts on health outcomes. We concur with Kawachi et al. (2005) in their suggestion that strengthening information infrastructure to measure and monitor disparities in class and race will be useful in eliminating health disparities.

Limitations exist with our data and our analyses. The data were obtained from the provincial dataset of hospitalizations, for which data collection may not be precise because of human error and because the data were collected for purposes other than use for this study. As well, observational studies cannot indicate causation. Each of our predictor variables was independently associated with injury risk and each would be correlated with each other. Multiple regression analysis, when finding the most efficient and compact model, eliminates closely correlated variables; therefore, some variables may be eliminated in favour of others which could be functionally important to understanding causation for the research questions under study.

Our analyses show that socioeconomic disadvantages, rural place of residence, and Aboriginal ethnicity itself independently contribute to increased injury risk. We can only speculate about the pathways, and whether they are direct or indirect. The reduction in disparity of injury is encouraging.
